# A feasibility pragmatic clinical trial of a primary care network exercise and education program for people with COPD

**DOI:** 10.1186/s40814-020-00705-y

**Published:** 2020-10-26

**Authors:** Kelsey M. T. Hurley, Anne-Marie Selzler, Wendy M. Rodgers, Michael K. Stickland

**Affiliations:** 1 Faculty of Kinesiology Sport and Recreation, University of Alberta, Edmonton, AB Canada; 2 Edmonton North Primary Care Network, Edmonton, AB Canada; 3 West Park Healthcare Centre, Toronto, ON Canada; 4 G.F. MacDonald Centre for Lung Health Covenant Health, Edmonton, AB Canada; 5 Division of Pulmonary Medicine, Department of Medicine, University of Alberta, Edmonton, AB T6G 2J3 Canada

**Keywords:** Pulmonary rehabilitation, Chronic obstructive pulmonary disease, Primary care, Self-management, Self-efficacy, Exercise, Physical activity

## Abstract

**Background:**

Pulmonary rehabilitation is an important component of chronic disease management in chronic obstructive pulmonary disease (COPD) and has been shown to improve shortness of breath, exercise capacity, quality of life, and decrease hospitalizations. However, pulmonary rehabilitation capacity is low. Primary care may be an effective method for delivering disease management services to this population. The objective of this feasibility pragmatic clinical trial was to evaluate enrollment and completion of a primary care network exercise and education program for people with COPD.

**Methods:**

COPD patients (*N* = 23; mean age = 65 ± 9 years; FEV1 = 68 ± 20% predicted) were recruited after referral to a primary care network exercise program in Edmonton, Alberta. Participants self-selected either an 8-week 16-session supervised exercise program or an 8-week unsupervised exercise program where they received three visits with an exercise specialist. Both groups self-selected education sessions with clinicians for disease management support. Referrals, completion, and program outcomes (physical activity, exercise capacity and health status) were measured before (T1), immediately after (T2), and 8 weeks following the program (T3).

**Results:**

Forty-three referrals were received in 10 months, where a minimum of 50 was required in order for the program to be considered feasible. Twenty-three participants provided baseline data, and twenty participants started the exercise program (10 in each exercise group), 16 of which completed the exercise program (80%). On average, 48% of the recommended education sessions were completed by participants.

**Conclusions:**

Enrollment into a COPD exercise and education program in a primary care network was low indicating the need for improved referral processes from physicians. Completion rates by participants were adequate for exercise but not education. The low referral rate and the lack of enrollment in COPD education by the patients indicate that a large-scale trial of the program as designed is not feasible.

## Key messages regarding feasibility

- What uncertainties existed regarding the feasibility? It was unknown whether there would be sufficient referrals to a COPD management program within a primary care network. The exercise and education program offered was different than traditional pulmonary rehabilitation, and it was unknown if patients would attend and complete this new program.
- What are the key feasibility findings? The program in its current form is not feasible due to low enrollment in the program and low completion of the education portion of the program. However, of those who began the program, completion rates for the exercise program were satisfactory.
- What are the implications for the feasibility findings for the design of the main study? The low referral rate and the lack of enrollment in COPD education by the patients indicate that a large-scale trial of the program as designed is not feasible prior to additional work to determine factors limiting referrals, enrollment, and adherence.

## Background

Chronic obstructive pulmonary disease (COPD) is characterized by shortness of breath and is the fourth leading cause of death in Canada [1]. COPD is a top reason for hospitalization and hospital readmission in those aged 60–74 [2] and is a substantial healthcare cost on society [3]. Improved management of COPD could lead to decreased hospitalization and cost to the healthcare system.

Due to the combination of exercise training, education, and patient support, pulmonary rehabilitation (PR) has been shown to improve shortness of breath, exercise capacity, quality of life, and decrease hospitalizations [4]. Unfortunately, capacity for PR programs in Canada is low, due to a lack of funding and program availability [5]. While hospitals continue to be the most common location to run PR programs, alternative settings such as primary care networks (PCNs) and community recreation centers have begun to offer these services [4, 5]. PCNs target health care needs through a network of family physicians in collaboration with health care teams that include a variety of healthcare professionals [6]. PCNs also serve the healthcare needs of patients within their own community allowing patients to access care closer to home. Additionally, home-based PR programs have been shown to be effective at improving quality of life and exercise capacity [7], and PCNs can provide exercise support for the development of home-based exercise programs. By understanding how different types of COPD management programs can be integrated into community-based programming, we may be able to help more COPD patients improve the long-term management of their disease and decrease hospital admissions and health care costs associated with COPD treatment and management [8].

While outcomes from PR are favorable, long-term adherence to exercise in people with COPD remains an issue [9, 10]. Self-efficacy, which is the confidence one has for performing a behavior [11], has been found to be associated with self-management behaviors such as exercise in people who have COPD [12, 13]. Self-efficacy can be measured using a domain-specific questionnaire. People with a strong sense of self-efficacy pursue their goals with persistent effort [11]. Evaluation of new programs in alternative settings, such as PCNs, should include motivational outcomes, such as self-efficacy, to identify potential improvements in long-term adherence.

Considering the lack of PR capacity and the goal of providing COPD management services closer to a patient’s home, the primary aim of this study was to evaluate the feasibility of a PCN exercise and education program for people with COPD, where patients can self-select the type of education and exercise program they receive (supervised vs. unsupervised). Feasibility was evaluated through enrollment in the program and completion of the full exercise and education protocol. Enrollment in the program was measured by patient referrals and self-selection of exercise and education. In order for the program to be considered feasible, a minimum of 50 referrals were required over 10 months, which is the value the PCN uses to determine the feasibility for integrating new programs. Completion was measured by patient attendance of both exercise and education sessions. As a secondary aim, this study also measured health outcomes before and after the program to help inform future power calculations and compared patient demographics with a local traditional PR program to determine if there are differences between patients who access these two programs.

## Methods

### Site and participants

This pragmatic study used data from an ongoing clinical evaluation of a COPD management program at the Edmonton North PCN. Patients were eligible for the study if they reported a diagnosis of COPD from their family physician or pulmonologist. Participants needed to be able to ambulate (with or without an aid), be free of unstable cardiovascular disease, be able to read and communicate in English, and not be currently engaging in any structured aerobic exercise. Excluded patients continued to receive usual care. All study procedures were approved by the University Health Research Ethics Board (Pro00070342) and by the Edmonton North PCN. The measures described below were collected as part of PCN program procedures. Patients provided written informed consent to participate in the study and for their de-identified data to be used for research purposes. To better understand the participants within the PCN program relative to traditional PR, baseline patient characteristics were compared to a cohort of patients attending the local traditional PR program in Edmonton who had also signed informed consent.

### Study design

This feasibility pragmatic clinical trial was conducted between March 2017 and April 2018. Patients referred for COPD disease management were contacted and scheduled for a telephone triage appointment with a PCN clinician during which patients were booked for group and/or individual appointments. Recruitment for the study occurred during their initial appointment with an exercise specialist, or during a COPD information group class, in which case they were booked for an initial appointment with an exercise specialist.

During their initial appointment with an exercise specialist (T1), baseline assessments occurred, which included exercise capacity, health status, and self-efficacy. Participants were provided an Omron-HJ324U accelerometer to record daily steps and an exercise diary to record aerobic exercise type, minutes, and intensity. Participants chose between supervised and unsupervised exercise programs and were booked appointments accordingly. There was no randomization to group as part of the goal of this feasibility study was to determine what type of program patients would choose to attend.

### COPD management program

The PCN offers self-management support for a variety of chronic conditions including COPD. For patients with COPD, the program consisted of COPD education, plus either a group or home exercise program. All COPD management education content used was from the Living Well with COPD [14] website and included (1) an introductory group session that discussed lung anatomy and pathophysiology of COPD and provided an overview of the health behaviors required to stay healthy; (2) individual inhaler review with a pharmacist; (3) meeting with an exercise specialist to learn about breathing management, coughing, and energy conservation and getting started with an exercise program; (4) creating an action plan for acute exacerbation of COPD (AECOPD); (5) meeting with a dietitian to discuss proper nutrition; (6) meeting with a mental health practitioner to learn how to manage anxiety and depression; and (7) smoking cessation with a tobacco educator (for current smokers). Patients chose which COPD management education sessions they wanted to attend; therefore, in some cases, patients did not attend all sessions. To ensure those with financial limitations could access care, patients had access to the PCN compassion fund for public transit passes if necessary.

The supervised exercise program consisted of 16 classes over 8 weeks and required participants to attend two times per week for 90 min each. Groups contained a maximum of eight participants each with one exercise specialist leading the classes. Classes were a mix of participants referred for a variety of conditions (e.g., COPD, cardiovascular disease, diabetes, obesity, chronic pain); therefore, exercise content varied for each participant based on their reason for referral. The program was run at a community recreation facility. Participants were asked to provide cost recovery for admission passes into the recreation facility, if financially feasible ($90–100 for the 8-week program). Participants who were classified as low income were assisted with a free or subsidized membership to access the facility throughout the sessions. The unsupervised exercise program consisted of three appointments with an exercise specialist at the PCN. The appointments were scheduled 2 to 3 weeks apart over approximately 8 weeks. Each appointment was 60 min long. The exercise specialist demonstrated exercise technique, provided instruction to use equipment, and used motivational interviewing techniques and goal setting to assist patients in creating an individualized exercise plan.

Participants in both exercise programs were encouraged to work up to the published exercise recommendations for people with COPD of 150 min per week of aerobic activity, accumulating this time in bouts of 10 min or more, and one to three sets of eight to twelve repetitions of four to six different resistance exercises two to three times per week [15]. In the unsupervised exercise group, the mode of aerobic exercise was based on preferences and equipment available. Participants in the supervised exercise group had many modes of exercise to choose from based on equipment availability in the community recreation center (e.g., treadmill, stationary bike, elliptical). In both groups, exercise intensity was prescribed by the exercise specialist using the rating of perceived exertion (RPE) scale for breathlessness and fatigue [16], with the goal for patients to exercise at four (somewhat severe) to six (more severe) out of ten (maximal). Intensity was monitored by the exercise specialist in the supervised exercise sessions and self-monitored in the unsupervised exercise sessions. Intensity was recorded by the participant on their exercise diary in both exercise groups.

### Program outcomes

Baseline average daily step count was recorded from their accelerometer on the first day of their supervised exercise program or during their next visit with the exercise specialist (unsupervised exercise program). Post-program measurements (T2) were completed at the end of the 8-week exercise program and included exercise capacity, health status, self-efficacy, average steps per day, and weekly aerobic exercise minutes. Follow-up measurements (T3) were completed 8 weeks after the exercise program was complete (i.e., 16 weeks) and consisted of the same assessments at T2.

### Patient comparison between programs

Baseline characteristics (age, sex, BMI, smoking history, lung function, dyspnea, quality of life, and self-efficacy, as described below) of COPD patients attending the PCN exercise and education program and COPD patients attending the local traditional PR program were obtained from clinical health records.

### Measures

#### Demographics

Age, sex, body mass index (BMI), smoking history, comorbidities, socioeconomic status (SES), education level, employment status, and marital status were collected through the patients’ electronic medical files and through questionnaires.

#### Lung function

Forced expiratory volume in 1 second (FEV1) and forced vital capacity (FVC) were collected through pre-program spirometry. A ratio of FEV1/FVC < 0.7 after bronchodilator confirmed COPD [17], and severity was categorized based on GOLD [17].

#### Modified medical research council (mMRC) dyspnea scale

The mMRC dyspnea scale was used to identify symptoms of breathlessness on a 5-point scale [18].

#### Attendance

The number of exercise and education sessions attended was tracked through electronic medical records. Completion of the exercise program was defined as attending at least 67% of the exercise sessions and the post-program assessment.

#### Health status

The COPD assessment test (CAT) is an eight-item questionnaire that scores participants on a 0–40 range with a lower score indicating less impairment and high score indicating more impairment. The CAT is a widely used questionnaire that has been shown to be valid and reliable for measuring health status in COPD participants [19].

#### Exercise capacity

The 6MWT was conducted according to the ATS guidelines [20] and the distance (in meters) was used to assess exercise capacity.

#### Steps per day

The Omron-HJ324U was used to track daily steps. This accelerometer is a tri-axis accelerometer with a built in 7-day memory that was accessible to the participants from the device [21]. An average of 7-day step count was used for analyses.

#### Aerobic exercise

Participants used an exercise diary to record the mode of aerobic exercise, the minutes they performed the aerobic exercise, and their average RPE during the aerobic activity.

#### Self-efficacy for managing breathlessness

Three questions from the COPD self-efficacy scale (CSES) developed by Wigal et al. [22] were used to assess self-efficacy for managing breathlessness during exertion [12].

### Statistical analysis

Statistical analyses were performed using IBM SPSS Statistics 24. Univariate ANOVAs were run on each continuous variable, and chi-square analyses were run on all categorical variables to identify any baseline group differences. Means and standard deviations are reported for outcome data. The results reported are from an intention to treat analysis. To determine differences in baseline patient characteristics and outcome measures between the PCN exercise and education program and the local traditional PR program, one-way ANOVAs were performed for continuous variables and chi-square tests were performed for categorical variables.

## Results

### Participants

Pre-program steps per day was significantly different between supervised and unsupervised exercise groups, *F*(1,17) = 5.08, *p* = .038, *η*^2^_*p*_ = .23, and employment status was significantly different between participants in the supervised exercise and unsupervised exercise groups, *χ*^2^ (1, *N* = 23) = 7.30, *p* = .01, with participants in the unsupervised exercise group being more active and more likely to be employed (Table 1).

**Table 1:** Baseline demographic descriptive statistics by group

	Supervised exercise	Unsupervised exercise	Between-group difference
	M (SD)	M (SD)	*p* value
Age, years	66.92 (9.05)	61.50 (9.62)	.180
Sex, % female	69.20	70.00	.968
BMI, kg/m^2^	33.72 (7.58)	35.72 (9.38)	.613
Pack years smoking, years	43.17 (18.01)	49.22 (38.56)	.636
Smoking history, % smoking	38.50	10.00	.123
Marital status, % married	30.80	30.00	.968
Education, % less than high school	7.70	10.00	.846
Employment, % working	7.70	60.00*	.007
History of PR, %	33.30	10.00	.193
Comorbidities, % with 2 or more	76.90	50.00	.570
Supplemental oxygen, %	15.40	0.00	.194
Referral type, % external	46.20	40.00	.768
mMRC dyspnea, 0–4	1.83	1.70	.787
FEV1 % predicted	61.64 (21.15)	76.50 (16.20)	.115
FEV1/FVC	57.64 (15.81)	60.63 (10.50)	.649
Steps per day	2383 (1472)	4783 (2865)*	**.**038
Self-efficacy for Managing Breathlessness, %	45.00 (13.40)	52.00 (22.00)	.376
6MWT, m	369 (81)	430 (115)	.150
CAT Total score, 1–40	19.75 (6.27)	17.20 (5.77)	.337

*Note*. Supervised exercise *N* = 13, Unsupervised exercise *N* = 10

*BMI* body mass index, *PR* pulmonary rehabilitation, *mMRC* modified medical research council, *FEV1* forced expiratory volume in 1 second, *FVC* forced vital capacity, *6MWT* six-minute walk test, *CAT* COPD Assessment Test

*Significant difference between groups based on *p* < .05

### Feasibility

**Fig. 1:**
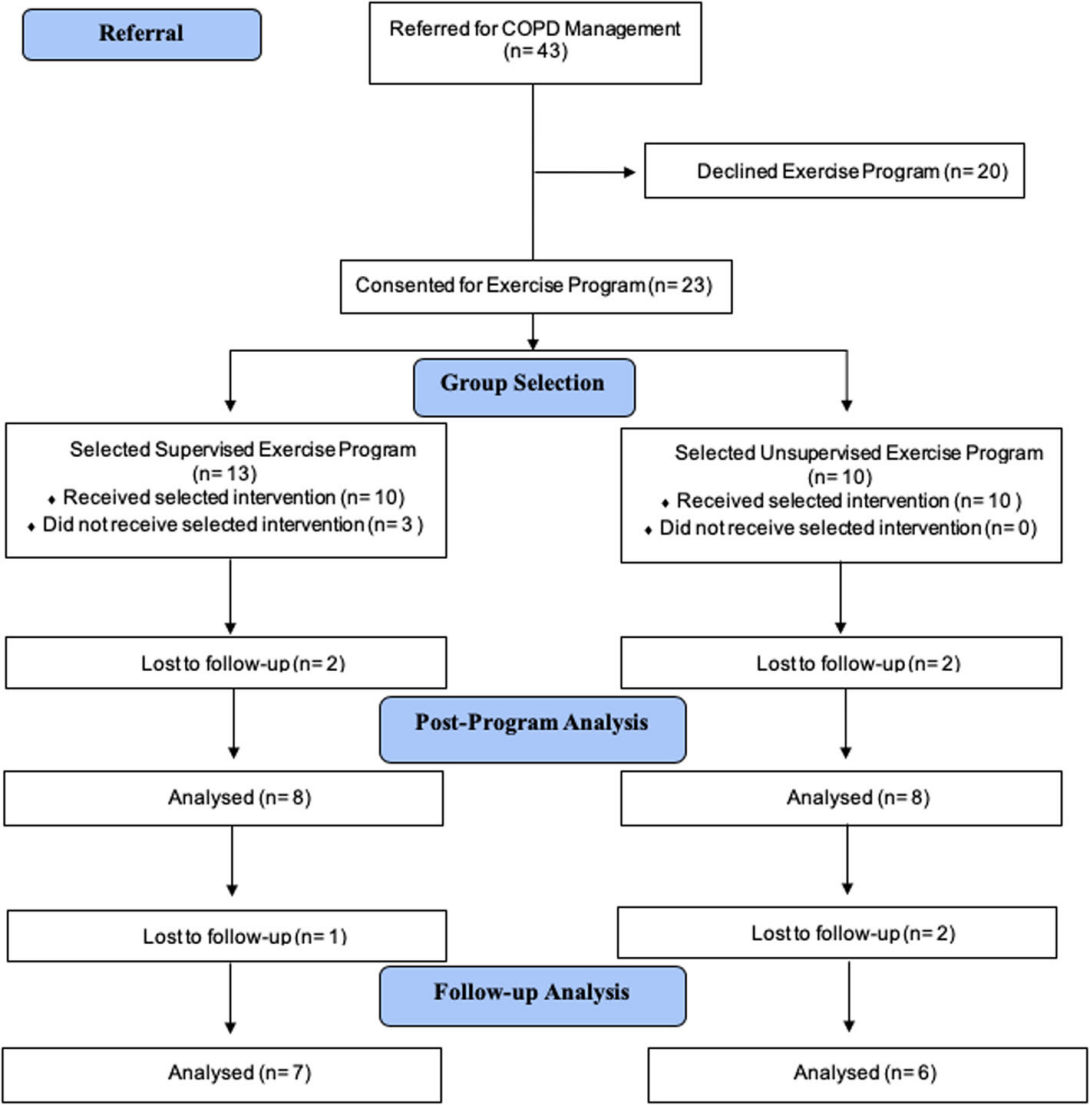
Referral to COPD management program and subsequent drop-out

Forty-three patients were referred to the PCN exercise and education program for COPD management support. Twenty-three patients consented to participate in the study. Descriptive statistics for all variables are summarized in Table 1. Thirteen patients choose the supervised exercise group and 10 choose the unsupervised exercise group. Drop-out is summarized in Fig. 1. Sixteen patients (80%) completed the exercise programs and provided post-program data. Participants completed a mean of 48% of recommended education sessions.

### Analysis of program outcomes

The means and standard deviations for all program outcomes are presented in Table 2. Our data suggest that health status, aerobic exercise, and self-efficacy for managing breathlessness improved similarly in both groups over the course of the 8-week exercise programs (T1 to T2) with no further improvement during the follow-up period (T2 to T3). There was no apparent improvement in exercise capacity or steps per day in either group over the course of the 8-week exercise programs (T1 to T2) or during the follow-up period (T2 to T3).

**Table 2:** Descriptive statistics of outcomes across time by group

	Supervised exercise			Unsupervised exercise		
	T1	T2	T3	T1	T2	T3
	M (SD)	M (SD)	M (SD)	M (SD)	M (SD)	M (SD)
CAT total score, 1-40	19.64 (6.02)	17.46 (6.49)	17.77 (6.83)	17.20 (5.77)	14.20 (5.07)	15.20 (6.03)
6MWT, m	369 (81)	376 (122)	369 (113)	430 (115)	449 (126)	446 (119)
Steps per day	2383 (1472)	2895 (1307)	2604 (1598)	4783 (2866)	5057 (2306)	4499 (2279)
Aerobic exercise minutes per week	0	37 (64)	44 (68)	0	86 (82)	104 (136)
Exercise RPE dyspnea, 1–10		3.7 (1.5)	2.6 (1.1)		3.4 (1.1)	3.5 (1.4)
6MWT RPE dyspnea, 1–10	4.5 (2.3)	5.1 (2.7)	4.2 (1.3)	4.5 (1.7)	3.9 (0.7)	4.4 (1.5)
Attendance, %		59.69 (38.59)			80.20 (17.04)	
Self-efficacy for managing breathlessness, %	45.00 (13.40)	49.60 (15.00)	50.60 (17.60)	53.80 (21.60)	61.20 (22.00)	65.60 (19.40)

*Note*. The data presented were calculated including the last value carried forward (intention to treat). Supervised exercise *N* = 13, Unsupervised exercise *N* = 10

*RPE* rating of perceived exertion, *T1* pre-program, *T2* end of program, *T3* 8 weeks after program completed, *6MWT* six-minute walk test, *CAT* COPD Assessment Test

### Patient comparison between programs

Means and standard deviations of patient demographics and outcome variables for the traditional PR and PCN samples are presented in Table 3. At baseline, there was a significant sex difference between samples, in that the PCN sample had more females than the traditional PR sample, *χ*^2^ (1, *N* = 333) = 3.96, *p* = .047. The PCN sample also had a significantly higher BMI than the traditional PR sample, *F*(1,328) = 7.008 , *p* = .009, *η*^2^_*p*_ = .021. The traditional PR sample had a significantly higher mMRC dyspnea than the PCN sample, *F*(1,312) = 30.13, *p* = .000, *η*^2^_*p*_ = .090, indicating that participants in the traditional PR program were more short of breath than participants in the PCN exercise and education program.

**Table 3:** Comparing PCN and traditional PR programs

	PCN		Traditional PR		Difference between samples
	*N*	Mean (SD)	*N*	Mean (SD)	*ρ* value
Age, years	23	64.57 (9.49)	311	65.37 (11.16)	.735
Sex, % female	23	69.57	311	48.1*	.047
BMI, kg/m^2^	19	34.56 (8.20)	311	29.77 (7.63)*	.009
Pack years smoking, years	21	45.76 (27.97)	248	40.46 (42.57)	.573
Currently smoking, %	23	26.10	302	18.2	.351
mMRC Dyspnea, 0–4	22	1.77 (1.11)	292	2.99 (.98)*	.000
FEV1 % predicted	19	67.89 (20.18)	293	60.74 (24.57)	.215
FEV1/FVC	19	58.89 (13.57)	293	53.86 (16.62)	.197
6MWT, m	23	396 (100)	284	371 (116)	.325
CAT total score, 1–40	23	18.58 (5.91)	295	19.52 (7.58)	.563
Steps per day	19	3646 (2566)	232	4596 (3184)	.206
Self-efficacy for managing breathlessness	22	49.91 ± 17.46	238	60.83 (28.26)	.076

*BMI* body mass index, *mMRC* modified medical research council, *FEV1* forced expiratory volume in 1 second, *FVC* forced vital capacity, *6MWT* six-minute walk test, *CAT* COPD Assessment Test

*Significant difference between PCN and traditional PR based on *p* < .05

## Discussion

This study examined the feasibility of a community-based COPD management program. The completion rate for the exercise portion of the program was 80%; however, lower than expected referrals and low completion of the education sessions suggest that the current referral process and some program details need to be altered to improve the feasibility of this type of PCN community-based intervention.

In regard to referrals, forty-three referrals were received in 10 months, where a minimum of 50 was required for the program to be considered feasible. During the year of data collection, 11,733 patients accessed chronic disease management services at the PCN, and of these patients, 74 reported COPD (0.6%). Interestingly, the prevalence of COPD in Alberta was estimated as 9.1% in 2015 [23]. This indicates a low referral rate of COPD patients to the PCN in general. It is unclear if this is due to patient willingness to report COPD as a comorbidity or low referrals from family physicians. This finding is not unique to the PCN; it has been reported that less than 10% of people with COPD are being referred to PR [24].

The literature strongly supports self-management education as a core component of COPD management [1, 25]. Within the PCN exercise and education program, COPD education was provided with a patient-centered approach, where patients could choose the education sessions they wanted to receive. Having patients self-select their education resulted in an average of 48% of recommended education sessions being completed. These findings suggest that providing patients the choice of which education sessions they want to attend may result in less education and self-management support than is ideal for effective COPD management. Completion rates for the PCN exercise program exceeded our expectations with 80% of participants completing the program. Typical dropout from PR has been reported at approximately 30% [26]. However, the local traditional PR program has reported 20% dropout [27]. Having patients exercise within their PCN may be an excellent site alternative as completion rates appear high.

As a secondary aim, health outcome data were collected in those who completed the trial. The overall sample size was very small, and care should be taken when evaluating these data; however, some interesting observations were observed. The supervised and unsupervised exercise groups did not meet the minimum clinically important difference (MCID) of 25–35 m for the 6MWT [28], which is typically observed following PR in patients with COPD. It is important to note that the exercise environment and intake process in the PCN is quite different than traditional PR, and this could impact the exercise prescription and ultimately the patients’ response to exercise. For instance, patients accessing the PCN program would not have received a maximal cardiopulmonary exercise test prior to beginning their program, and during the PCN exercise program, there is no access to supplemental oxygen or an emergency crash cart. This may contribute to exercise being prescribed at a lower intensity for a patient in a community recreation facility. Exercise prescribed for unsupervised exercise may be influenced by the same factors. The participants in the PCN exercise and education program also demonstrated no change in physical activity as evaluated by steps per day and participants did not achieve the recommended 150 min per week of aerobic exercise. A more aggressive exercise prescription by PCN exercise specialists is likely necessary to improve exercise capacity for people with COPD [29].

This study provides preliminary evidence that those who participate in a community PCN program may be different than those who access PR (as evaluated by baseline dyspnea, BMI, sex). These differences are important because low referral to PR could result in a substantial portion of the COPD population not having access to COPD management services. In cardiovascular disease management, this notion is supported where certain patient groups are less likely to access cardiac rehabilitation such as women, ethnocultural minority groups, and those with lower socioeconomic status [30]. Additionally, this study suggests that people with COPD who continue to work may be more likely to select unsupervised exercise and may be less likely to attend traditional PR. If these population differences are relevant in pulmonary disease management, simply increasing referral and availability of traditional PR programs may not improve participation for certain patient populations. Therefore, community-based COPD management, with more flexible disease management and exercise options, may be appropriate for a proportion of the population not accessing traditional PR. In regard to referral rate, 19% of PR programs across Canada reported limited effectiveness of current referral systems [5] and it is possible that having multiple sites and program selections could increase confusion of the referring physician. For this reason, as new PR sites are developed to provide better access, a central referral process could help decrease referral barriers to PR. In addition to this, exploring self-referral for patients, community advertising and improved communication between allied health professionals and physicians could all help to improve referral issues.

There are several limitations of this study. The sample size of the current study was small; therefore, preliminary outcomes regarding the effectiveness of the program should be interpreted with caution. Self-report of aerobic exercise was also a limitation. While we supplemented self-report with objective accelerometer data, we recognize that the two assessments were measuring different elements of physical activity (i.e., only walking activity vs. any aerobic exercise including walking) and it is possible that participants inflated their self-report of exercise in order to please their exercise specialist or researcher. Future studies should consider an accelerometer that measures multiple modes of aerobic exercise. Finally, the lack of randomization to groups resulted in baseline differences between groups which may limit interpretation of the results.

## Conclusion

This study provides preliminary evidence that a large-scale trial of a PCN exercise and education program for people with COPD in the current form is not feasible. However, PCNs should remain a potential community-based venue to offer COPD management support because they offer flexible exercise options and are accessible to a proportion of the COPD patient population who may not attend traditional PR. To improve enrollment feasibility, changes to the referral system should be implemented to ensure PCNs are receiving enough referrals to run consistent programming. Preliminary results suggest this style of program may not be as effective as traditional PR. Therefore, the education portion of the program should be designed to promote higher completion rates for disease self-management support, and the exercise program should include adequate quantity and intensity to promote improvements in exercise capacity.

## Supplementary Information

**Additional file 1.** PCN data set

**Additional file 2.** PCN vs. Breathe Easy Data Set

**Additional file 3.** CONSORT Abstract Checklist

**Additional file 4.** CONSORT Checklist

## Data Availability

All data generated and analyzed during the current study are included in this published article and its supplementary information files.

## Abbreviations

6MWT: Six-minute walk test
AECOPD: Acute exacerbation of chronic obstructive pulmonary disease
ANOVA: Analysis of variance
ATS: American Thoracic Society
BMI: Body mass index
CAT: COPD Assessment Test
COPD: Chronic obstructive pulmonary disease
CSES: COPD Self-efficacy Scale
FEV1: Forced expiratory volume in 1 second
FVC: Forced vital capacity
HRQOL: Health-related quality of life
MCID: Minimal clinically important difference
mMRC: Modified medical research council
PCN: Primary care network
PR: Pulmonary rehabilitation
RPE: Rating of perceived exertion
SES: Socioeconomic status
